# How cryoprotectants work: hydrogen-bonding in low-temperature vitrified solutions†

**DOI:** 10.1039/d2sc03188d

**Published:** 2022-08-08

**Authors:** Euihyun Lee, Carlos R. Baiz

**Affiliations:** Department of Chemistry, The University of Texas at Austin Austin TX 78712 USA cbaiz@cm.utexas.edu

## Abstract

Dimethyl sulfoxide (DMSO) increases cell and tissue viability at low temperatures and is commonly used as a cryoprotectant for cryogenic storage of biological materials. DMSO disorders the water hydrogen-bond networks and inhibits ice-crystal growth, though the specific DMSO interactions with water are difficult to characterize. In this study, we use a combination of Fourier Transform infrared spectroscopy (FTIR), molecular dynamics simulations, and vibrational frequency maps to characterize the temperature-dependent hydrogen bonding interactions of DMSO with water from 30 °C to −80 °C. Specifically, broad peaks in O–D stretch vibrational spectra of DMSO and deuterated water (HDO) cosolvent systems show that the hydrogen bond networks become increasingly disrupted compared to pure water. Simulations demonstrated that these disrupted hydrogen bond networks remain largely localized to the first hydration shell of DMSO, which explains the high DMSO concentrations needed to prevent ice crystal formation in cryopreservation applications.

## Abstract 2

Dimethyl sulfoxide (DMSO) increases cell and tissue viability at low temperatures and is commonly used as a cryoprotectant for cryogenic storage of biological materials.

## Graphical abstract

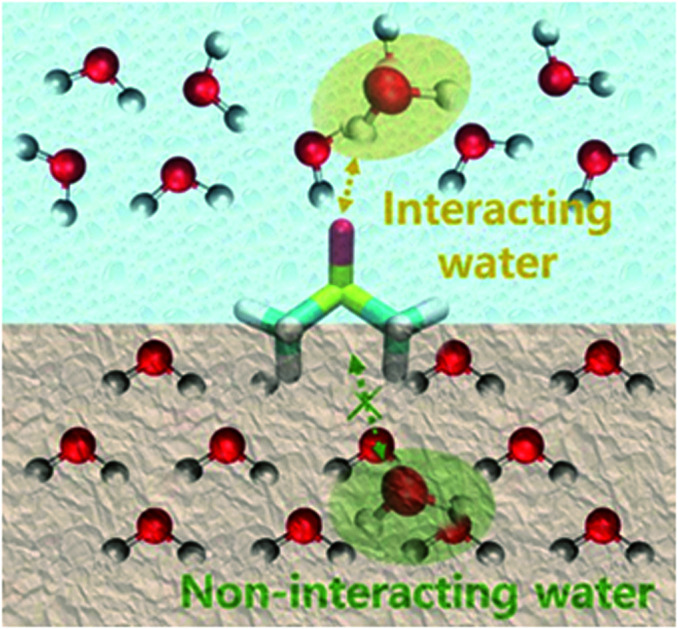

## Introduction

Currently, the only viable option for long-term cell and tissue storage is low-temperature cryopreservation. Mechanical shearing and osmotic stress resulting from ice crystal formation, can, however, damage biomolecules, cells, and tissues.^1,2^ Cryoprotectants are commonly used to inhibit ice crystal formation and increase cell viability during the freezing and thawing processes.^3–7^ DMSO is the most widely-used cryoprotectant, but its ice-inhibition mechanisms and interactions with water at low temperatures are not well understood. The strong polar nature of DMSO and lack of H-bond donors result in strong dipole–dipole and H-bond interactions with water. Interestingly, at room temperature, DMSO also forms nanometer-scale domains enriched in either DMSO or water as a result of microscopic liquid–liquid phase separation.^8^ Understanding the interactions of DMSO with liquid or vitrified water at low temperatures is important to obtain a molecular-level mechanistic understanding of the ice-crystal inhibition process. Here we characterize the structures and populations of H-bond ensembles in DMSO/water cosolvent systems from 30 to −80 °C using infrared absorption spectroscopy, and molecular dynamics simulations.

**Fig. 1:**
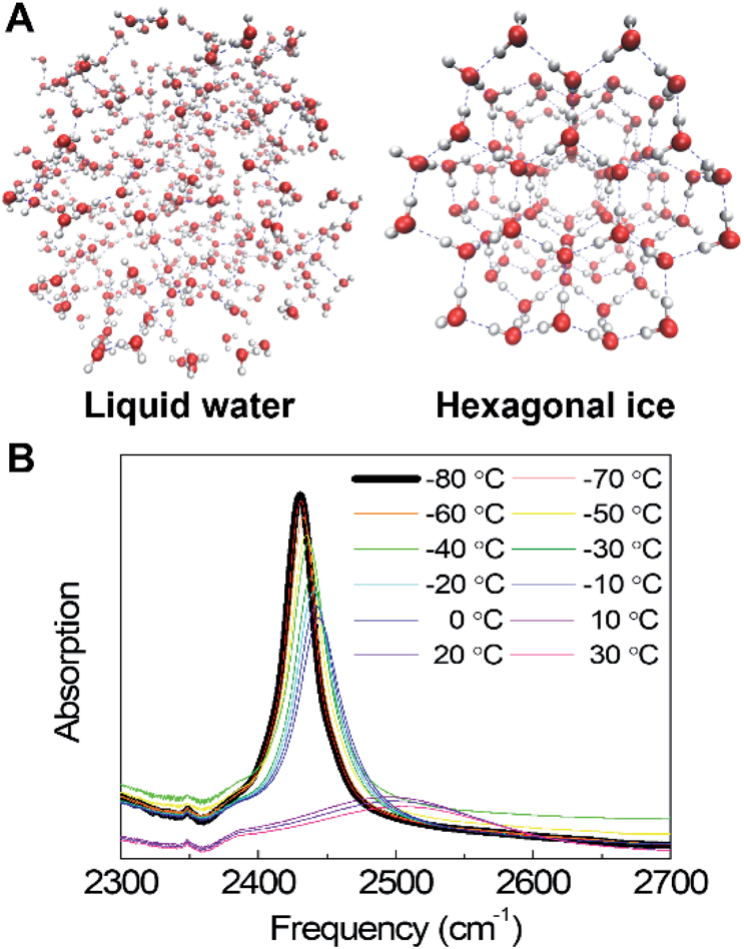
(A) Schematic representation of the liquid water structure and the crystalline water structure. (B) Temperature-dependent IR absorption spectra of the O–D stretching mode of dilute deuterated water (HDO) in H_2_O (pure water system). The small feature near 2350 cm^−1^ arises from incomplete background subtraction of CO_2_ gas in the sample enclosure.

Vibrational spectroscopy is an ideal tool to measure H-bond configurations in liquid and crystalline phases, as the O–H (or O–D) stretching frequency is highly sensitive to the local electrostatic environment;^9–14^ as a consequence, strong H-bonds result in a red-shifted peak whereas weak or bifurcated H-bonds result in more blue-shifted frequencies. Ice *I*_h_ (hexagonal ice) is defined by near-perfect tetrahedral H-bond networks, which produce narrow bands in the spectrum, whereas increased heterogeneity and weaker H-bonds in liquid water result in a significantly broader and blue-shifted band (Fig. 1). Tracking the frequencies, intensities, and lineshapes of the water vibrational modes across a range of DMSO concentrations reports on direct DMSO-water on H-bonding interactions as well as the disruption of the H-bond network. Molecular dynamics (MD) simulations produce atomistic-level descriptions of H-bonding populations and geometries and have been extensively benchmarked against experiments for these systems by comparing computed frequencies, lineshapes, and timescales with measured FTIR and 2D IR spectra across a wide range of concentrations.^15–17^ In this study, simulations produce a good qualitative agreement with experiments, demonstrating that modern MD force fields accurately model DMSO–water interactions across a wide temperature range. In summary, measuring the structure of water in cryopreservation solutions is important to explore the ice-crystal-inhibition mechanisms, and the combination of IR spectroscopy and simulations provides a detailed molecular-level picture.

## Methods

### A. Sample preparation

Dimethyl sulfoxide (DMSO) was purchased from Fisher Scientific (≥99.7%), and deuterium oxide (D_2_O) was purchased from Cambridge Isotope Laboratories, Inc. (99.9% D). All chemicals were stored under a dry atmosphere and used without further purification. 0, 11, 22, and 33 wt% DMSO mixtures consisted of DMSO and 5 wt% D_2_O in ultrapure Milli-Q H_2_O.

### B. FTIR spectroscopy

FTIR spectra of DMSO cosolvent systems were measured at 0.5 cm^−1^ resolution using a Bruker Vertex 70 FTIR spectrometer. The spectrometer was purged with dry air to remove absorption peaks from water vapor. The small feature around 2350 cm^−1^ is the result of incomplete subtraction of CO_2_ gas in the sample enclosure. Each spectrum is an average of 32 scans from 1000 to 4000 cm^−1^. The sample was equilibrated for 5 minutes at each temperature setpoint, using a Specac cryostat (WEST P6100) with a vacuum pump and using liquid nitrogen as the coolant. Spectra were measured at the following temperatures: −80, −70, −60, −50, −40, −30, −20, −10, 0, 10, 20, 30 °C. Experiments were repeated to ensure the reproducibility of measured lineshapes. The peak fitting procedure is described in Section S1.†

### C. Molecular dynamics simulations

Simulations were performed using the GROMACS2019.4 simulation package^18^ and the CHARMM36 general force field.^19^ Three systems were constructed for comparison with experiment: (1) pure water at 298 K, (2) pure water at 198 K, and (3) 33 wt% DMSO solution at 198 K. The temperature-dependence of vibrational frequencies in pure water was observed by comparing (1) and (2) as shown in Fig. S2.† The effect of DMSO on the vibrational frequencies was observed by comparing (2) and (3) as shown in Fig. S2.† The initial random configurations of pure water and DMSO solution were constructed using the Packmol package.^20^ The TIP4Pew water model was used for a room-temperature (298 K) simulation of pure water,^21^ and the TIP4P/Ice water model was used for a low-temperature (198 K) simulation of both pure water and DMSO cosolvent systems.^22^

Initial configurations of systems were energy-minimized for 10 000 steps. Next, a 50 ns NPT equilibration was performed to adjust a box density using the V-rescale thermostat^23^ at both 298 K and 198 K and the Berendsen barostat^24^ at 1 bar. Following the NPT equilibration, a 10 ns NVT equilibration was carried out using the V-rescale thermostat at 298 K and 198 K, respectively. Finally, the NPT production simulations were performed using the Nose–Hoover thermostat and the Parrinello-Raman barostat. Finally, 20 ns production trajectories were carried out with snapshots stored every 1 ps for analysis of structural ensembles and frequency distributions. Tetrahedral order parameters for the 33 wt% DMSO and pure water simulations at 198 K were calculated using the definition introduced by Debenedetti and coworkers.^25^

## Results and discussion

### A. Temperature dependence of the water vibrational mode

Temperature-dependent O–D stretching absorption spectra of dilute HDO in H_2_O in the (Fig. 1B) show that in ice, the spectrum is dominated by a single sharp band, which results from the localized O–D stretch, making the interpretation relatively simple compared to the coupled symmetric and asymmetric O–H (or O–D) stretches of pure H_2_O or D_2_O, which are delocalized over hundreds of molecules.^9,26,27^ Spectra show significant changes across the liquid-to-ice phase transition at the melting point. The ice spectrum is significantly narrower and red-shifted compared to the liquid water spectrum as a result of the highly regular tetrahedral H-bond networks. These tetrahedral structures become more disordered, and the H-bond configurations become increasingly heterogeneous, leading to a significantly broader band in the liquid phase. In both liquid water and ice, the spectrum becomes narrower and red-shifted with decreasing temperature as a result of decreasing structural disorder.^9,28^ In the presence of DMSO up to 33 wt%, the same general spectral changes are observed (Fig. S1†). However, unlike pure water where the solid–liquid phase transition is sharp, DMSO shows a smoother phase transition, indicating that DMSO disrupts the H-bond networks, creating a more disordered environment that leads to a broader melting transition. In summary, the temperature-dependent spectra of water show pronounced changes through the liquid-to-solid phase transition. FTIR spectroscopy then measures the temperature-dependent effects of DMSO in the liquid and solid phases.

### B. New spectral feature in DMSO/water cosolvent mixtures

In order to quantify the amplitudes, line widths (FWHM), and frequencies of the lineshapes in pure water and DMSO cosolvent systems, we performed a multi-component Gaussian fit of the measured spectra. In brief, the spectrum of pure water can be well-represented by a combination of three narrow Gaussian peaks, one high amplitude peak flanked by two low amplitude peaks of approximately equal width. The two observed sidebands originate from modulation of the O–D stretch by the low-frequency lattice modes of the crystal.^9,29^ The frequencies and amplitudes of these three peaks remain nearly identical in the DMSO solutions (Table S1†).

**Fig. 2:**
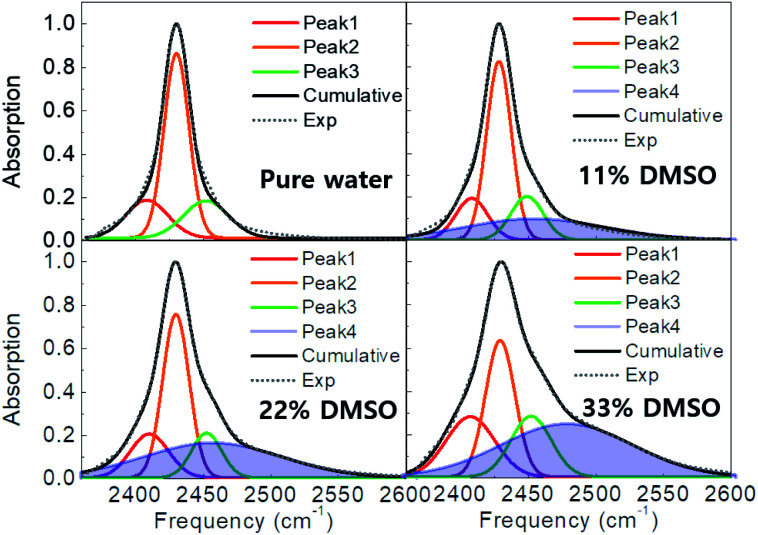
Measured FTIR spectra of pure water and water-DMSO cosolvent systems at −80 °C (dashed curves). The spectra are well-reproduced by a combination of Gaussian peaks, which are shown individually along with their cumulative sum (black curves). Here, peak shaded in blue represents the new peak, only present in the DMSO cosolvent systems. The area of this new peak increases with increasing DMSO concentration.

Interestingly, spectra of the DMSO solutions include an additional broad peak around 2455–2480 cm^−1^, which is indicated as a shaded area in Fig. 2. The width of this new peak is ∼120 cm^−1^, which is 3–4× broader than the peaks observed in pure ice, suggesting that this additional peak is associated with highly-disordered water H-bond configurations. The new peak area increases with higher DMSO concentrations (Table S1†), whereas the frequency and width remain approximately unchanged with DMSO concentration between 11 and 22 wt% but a further blue shift of 21 cm^−1^, from 2458 to 2479 cm^−1^, is observed at 33 wt% DMSO. Together, these observations indicate that the additional feature is associated with disordered—vitrified— water induced by the presence of DMSO.

### C. Simulations reveal origin of the new spectral feature

The origin of the observed spectral feature in the cosolvent systems can be interpreted by comparing experimental spectra with frequency distributions computed from MD snapshots and a vibrational frequency map for the O–D stretching mode.^30,31^ The simulations are described in Section S2,† in brief, three simulations were carried out: 1. pure water at 298 K; 2. pure water at 198 K; and 3. DMSO (33 wt%) at 198 K (−75 °C) and snapshots were sampled from the production trajectory. Next, HDO O–D stretch instantaneous frequency distributions across the three systems were computed using the vibrational map developed by Choi *et al.* (Table S2†),^31^ which converts the electrostatic potential at the H, O, and D atoms into a frequency shift. This enabled a one-to-one connection between local H-bond structures and O–D frequencies. Computed frequency distributions qualitatively reproduce the trends observed in the experimental spectra (Fig. S2†). In the low-temperature ice phase, the spectral shape becomes narrower and red-shifted compared to liquid water at ambient temperature. This indicates that simulations reproduce the strong, well-defined H-bond interactions, and periodic geometries of ice as indicated by radial and orientational distribution functions (Fig. S3†).^32,33^ The instantaneous frequency histograms of pure water and DMSO cosolvent system at 198 K show that the DMSO solution includes a blue-shifted and broader frequency distribution, suggesting the presence of DMSO results in a broader range of H-bond configurations (Fig. S2†). However, the bulk spectrum alone does not reveal the interactions leading to the additional H-bond ensembles. The radial and orientational distribution functions of water in the presence of DMSO do not show significant changes (Fig. S3†). Suggesting that increased heterogeneity is associated with water molecules within the DMSO first solvation shell as a result of short-range interactions, and the bulk liquid may not be affected.

**Fig. 3:**
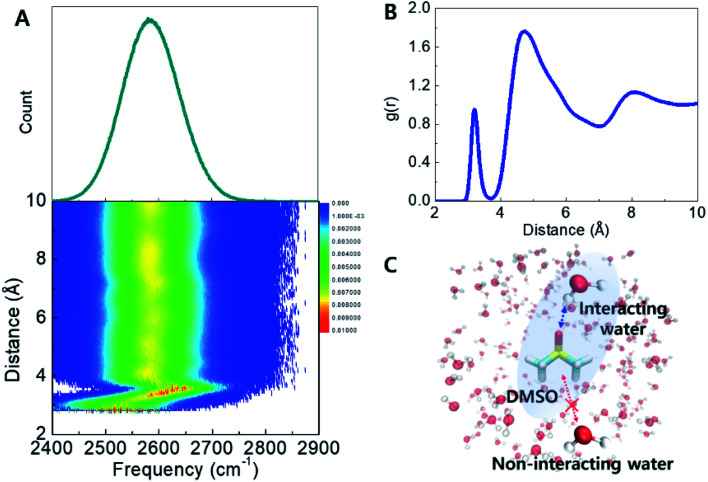
Instantaneous frequency distribution of the O–D stretch mode of HDO in 33 wt% DMSO solution at 198 K (top) and its effective 2D population with frequency and distance between S atom of DMSO and D atom of HDO (bottom). B. Radial distribution function of water hydrogens near DMSO sulfur atoms showing that the first solvation shell appears below a cutoff of 3.6 Å. C. Schematic representation of DMSO-interacting/non-interacting water.

The effects of DMSO on the H-bond networks are revealed in the two-dimensional histogram showing the population of water molecules as a function of both frequency and distance to DMSO (Fig. 3A). In this plot, *P*(*ω*,*r*), represents the average number of water molecules with frequency *ω* located at distance *r* from DMSO (S atom), in the unit shell having a thickness of *dr*. Interestingly, two features were revealed from this histogram. (1) The frequency distribution above a distance ∼3.6 Å is largely unchanged with increasing DMSO-water distance. This population dominates the computed frequency distribution. (2) Interestingly, at distances shorter distances than ∼3.6 Å there is a broad frequency distribution (see detail in Fig. S5†). This corresponds to waters within the first hydration shell (Fig. 3B). Together, this analysis reveals that water molecules in the first hydration shell of DMSO strongly contribute to the broad, high-frequency distribution observed in the experimental spectra (Fig. 2). In other words, the simulations suggest that the measured broad feature arises directly from water molecules directly interacting with DMSO, and therefore explains why the peak area increases proportionally with DMSO concentration.

Structural order parameters have been useful to quantify the tetrahedral ordering of water molecules across a range of temperatures both in the bulk as well as in solutions and complex cosolvent mixtures.^34–37^ Specifically, the tetrahedral order parameter has been useful as a means to quantify the ice-like ordered structure in supercooled water.^25^ The order parameter takes on a value of one for perfectly crystalline ice, and an average value of zero for a perfectly random gas. Here we compute the order parameter for waters within the first solvation shell of DMSO, as well as water molecules outside this first solvation shell. Fig. S6† shows histograms of the order parameter indicating that water molecules within the first solvation shell are significantly disordered, as shown by the lower values of *q*, but water molecules beyond the first solvation shell have a bulk-like geometry. This analysis confirms that the perturbation of DMSO is confined to its first solvation shell.

Together, the structural and frequency analysis suggests that in the DMSO solutions, a large fraction of water molecules (bulk water) adopts an ice-like configuration, but the interacting waters within the first solvation shell of DMSO produce relatively disordered configurations, as evidenced by the broad frequency distributions (Fig. S3†) and the structural order parameter (Fig. S6†). This provides an exciting hypothesis for how DMSO is able to disrupt ice-crystal formation by stabilizing disordered configurations which lack proper orientation to be incorporated into the ice lattice. This is the main mechanism by which DMSO can be used to vitrify water at relatively modest cooling rates.^4,38^

## Conclusions

In this work, we investigated how the crystalline water structure of hexagonal ice is affected by DMSO, a widely-used cryoprotectant. The studies were carried out using FTIR spectroscopy of dilute HDO in H_2_O/DMSO cosolvent systems accompanied by MD simulations of the same systems. Temperature-dependent FTIR spectra showed that narrower and more red-shifted peaks appear at low temperatures due to more ordered and rigid H-bond structures. In addition, concentration-dependent spectra revealed that, in the DMSO cosolvent systems, the HDO O–D vibration has a significantly broader absorption, compared with the pure water, indicating that DMSO disorders the H-bond networks (Fig. 2). Interestingly, we found that an additional high-frequency peak appears in DMSO cosolvent systems, and this is prominent at 33 wt% DMSO. Joint structural and frequency analysis of MD simulations revealed the origin of the new spectral feature. In brief, water can be separated into two structural ensembles based on whether a molecule is part of the first solvation shell of DMSO or not. Based on the broad FTIR peak and the result of simulations we conclude that water surrounding DMSO is highly disordered. Secondly, the results show that water not interacting with DMSO has a partially disrupted H-bond structure, and contributes to the dominant peak of the O–D vibration in DMSO cosolvent systems. These studies revealed the precise mechanism of how DMSO acts as a cryoprotectant by disrupting the tetrahedral H-bond structure, by disordering water molecules within its first hydration shell. Surprisingly, these interactions are very short range, which explains the large amounts of DMSO required for vitrification at moderate cooling rates.^4,38–41^ Interestingly, DMSO is known to undergo microscopic liquid–liquid phase separation to form nanometer-size clusters of DMSO and clusters of water containing hundreds of molecules.^8^ This suggests that within the clusters, only waters that directly interact with DMSO are affected whereas the core of the cluster remains unaffected. Ice crystal growth may not be possible if a significant fraction of the molecules is “locked” into a disordered configuration. This is an exciting hypothesis for how DMSO can prevent ice-crystal formation at low temperatures.

## Author contributions

Conceptualization, C. R. B.; simulations and experiments, E. L.; analysis, E. L. and C. R. B.; manuscript writing, E. L. and C. R. B.

## Conflicts of interest

There are no conflicts to declare.

## Data availability

Measured FTIR spectra, peaks fits, snapshots and input files necessary for reproducing production MD simulations can be downloaded from the Texas Data Repository (doi: 10.18738/T8/W7CVCA).

## Supplementary Material

SC-013-D2SC03188D-s001

## References

[cit1] Giwa S., Lewis J. K., Alvarez L., Langer R., Roth A. E., Church G. M., Markmann J. F., Sachs D. H., Chandraker A., Wertheim J. A. (2017). Nat. Biotechnol..

[cit2] Fahy G. M., Wowk B., Wu J., Phan J., Rasch C., Chang A., Zendejas E. (2004). Cryobiology.

[cit3] Lovelock J. E., Bishop M. W. (1959). Nature.

[cit4] Rall W. F., Fahy G. M. (1985). Nature.

[cit5] Fahy G. M. (2010). Cryobiology.

[cit6] Bakhach J. (2009). Organogenesis.

[cit7] Mazur P., Rall W. F., Leibo S. P. (1984). Cell Biophys..

[cit8] Oh K. I., You X., Flanagan J. C., Baiz C. R. (2020). J. Phys. Chem. Lett..

[cit9] Li F., Skinner J. L. (2010). J. Chem. Phys..

[cit10] Bertie J. E., Whalley E. (1964). J. Chem. Phys..

[cit11] De Marco L., Carpenter W., Liu H., Biswas R., Bowman J. M., Tokmakoff A. (2016). J. Phys. Chem. Lett..

[cit12] Choi J. H., Cho M. (2013). J. Chem. Phys..

[cit13] Yan C., Xue Z., Zhao W., Wang J., Mu T. (2016). Chemphyschem.

[cit14] Caporaletti F., Bonn D., Woutersen S. (2021). J. Phys. Chem. Lett..

[cit15] Oh K. I., Rajesh K., Stanton J. F., Baiz C. R. (2017). Angew. Chem., Int. Ed. Engl..

[cit16] Oh K. I., Baiz C. R. (2018). J. Phys. Chem. B.

[cit17] Kashid S. M., Jin G. Y., Bagchi S., Kim Y. S. (2015). J. Phys. Chem. B.

[cit18] Berendsen H. J. C., Vanderspoel D., Vandrunen R. (1995). Comput. Phys. Commun..

[cit19] Klauda J. B., Venable R. M., Freites J. A., O'Connor J. W., Tobias D. J., Mondragon-Ramirez C., Vorobyov I., MacKerell, Jr. A. D., Pastor R. W. (2010). J. Phys. Chem. B.

[cit20] Martinez L., Andrade R., Birgin E. G., Martinez J. M. (2009). J. Comput. Chem..

[cit21] Horn H. W., Swope W. C., Pitera J. W., Madura J. D., Dick T. J., Hura G. L., Head-Gordon T. (2004). J. Chem. Phys..

[cit22] Abascal J. L., Sanz E., Garcia Fernandez R., Vega C. (2005). J. Chem. Phys..

[cit23] Bussi G., Donadio D., Parrinello M. (2007). J. Chem. Phys..

[cit24] Berendsen H. J. C., Postma J. P. M., Gunsteren W. F. v., DiNola A., Haak J. R. (1984). J. Chem. Phys..

[cit25] Errington J. R., Debenedetti P. G. (2001). Nature.

[cit26] Rice S. A., Bergren M. S., Belch A. C., Nielsen G. (1983). J. Phys. Chem..

[cit27] Auer B. M., Skinner J. L. (2008). J. Chem. Phys..

[cit28] Shi L., Gruenbaum S. M., Skinner J. L. (2012). J. Phys. Chem. B.

[cit29] Gálvez Ó., Maté B., Herrero V. J., Escribano R. (2011). Astrophys. J..

[cit30] Baiz C. R., Blasiak B., Bredenbeck J., Cho M., Choi J. H., Corcelli S. A., Dijkstra A. G., Feng C. J., Garrett-Roe S., Ge N. H., Hanson-Heine M. W. D., Hirst J. D., Jansen T. L. C., Kwac K., Kubarych K. J., Londergan C. H., Maekawa H., Reppert M., Saito S., Roy S., Skinner J. L., Stock G., Straub J. E., Thielges M. C., Tominaga K., Tokmakoff A., Torii H., Wang L., Webb L. J., Zanni M. T. (2020). Chem. Rev..

[cit31] Choi J. H., Kim H., Kim S., Lim S., Chon B., Cho M. (2015). J. Chem. Phys..

[cit32] Kirkwood J. G., Boggs E. M. (1942). J. Chem. Phys..

[cit33] Lazaridis T., Karplus M. (1996). J. Chem. Phys..

[cit34] Tanaka H., Tong H., Shi R., Russo J. (2019). Nat. Rev. Phys..

[cit35] Russo J., Tanaka H. (2014). Nat. Commun..

[cit36] Duboué-Dijon E., Laage D. (2015). J. Phys. Chem. B.

[cit37] Oh K.-I., Baiz C. R. (2020). J. Chem. Phys..

[cit38] Fahy G. M., MacFarlane D., Angell C. A., Meryman H. (1984). Cryobiology.

[cit39] Fahy G. M., Lilley T. H., Linsdell H., Douglas M. S., Meryman H. T. (1990). Cryobiology.

[cit40] Wowk B., Leitl E., Rasch C. M., Mesbah-Karimi N., Harris S. B., Fahy G. M. (2000). Cryobiology.

[cit41] Fahy G. M., Wowk B., Wu J., Paynter S. (2004). Cryobiology.

