# Supplementary material for: How cryoprotectants work: hydrogen-bonding in low-temperature vitrified solutions
Source: Chem Sci. 2022 Aug 8;13(34):9980–4. doi: 10.1039/d2sc03188d (PMC9430440; doi:10.1039/d2sc03188d)
Supplement: SC-013-D2SC03188D-s001 [file SC-013-D2SC03188D-s001.pdf]

Electronic Supplementary Material (ESI) for Chemical Science.
This journal is © The Royal Society of Chemistry 2022

Electronic Supplementary Information (ESI) for

How cryoprotectants work: hydrogen-bonding in low-temperature vitrified solutions

Euihyun Lee[a] and Carlos R. Baiz*[a]

[a] *Department of Chemistry, the University of Texas at Austin, Austin, TX, USA*

** E-mail: cbaiz@cm.utexas.edu*

Section S1. Experimental Spectra and Peak Fitting

Gaussian Peak fitting was performed using the Peak Analyzer tool as implemented in OriginPro ver. 8.5 (Origin Lab Corp). The three-peak description of the spectrum was selected based on prior literature as discussed in the main text. The initial frequencies of the peaks was estimated based on a second-derivative analysis of the FTIR spectra, and these values were verified as the centers of the Gaussian peaks in the output fit. A fourth peak was included in the DMSO solutions to reproduce the broad feature described in the text (Figure 2, main text). Peak frequencies and amplitudes remain consistent across DMSO concentrations (Table 1), which ensured the consistency of the fits. The fits were performed using the Levenberg-Marquardt (L-M) algorithm which is an iterative procedure combining the Gauss-Newton method with the steepest descent method, the number of L-M iterations needed for convergence was 3 for the pure-water spectrum and 8 for the DMSO spectra. Raw spectra and fitted curves, together the inputs for MD simulations can be downloaded from the Texas Data Repository (https://doi.org/10.18738/T8/W7CVCA).

Figure S1. Temperature-dependent FTIR spectra of the OD stretch mode of HDO at the three different DMSO concentrations studied here. The concentration is indicated above each plot. The small feature near 2350 cm^{-1} is the result of incomplete background subtraction of CO_2 gas present in the sample chamber.

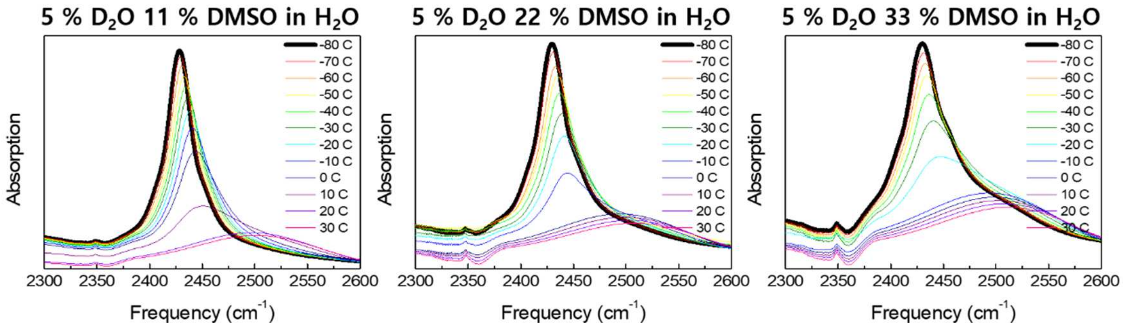

Figure S2. Computed instantaneous frequency distributions of the OD stretch mode of HDO in the three different systems. Inset represents experimentally measured FTIR spectra of water in pure water and 33 wt% DMSO solution at -80 ℃ (Figure 2, main text).

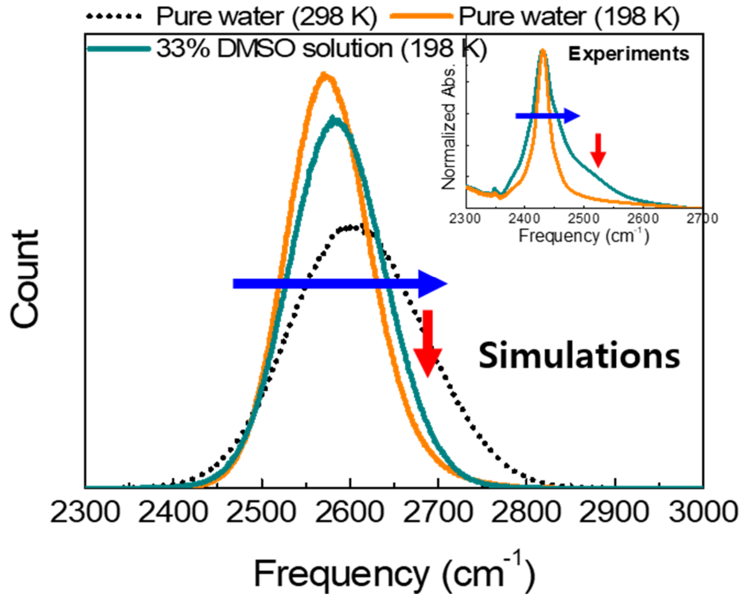

Figure S3. Computed water(O)-water(H) radial distribution functions and water OH vector orientational distribution functions from MD snapshots. Dotted line represents the pure water system at room temperature. Solid lines correspond to crystalline water systems with (dark cyan)/without (orange) DMSO.

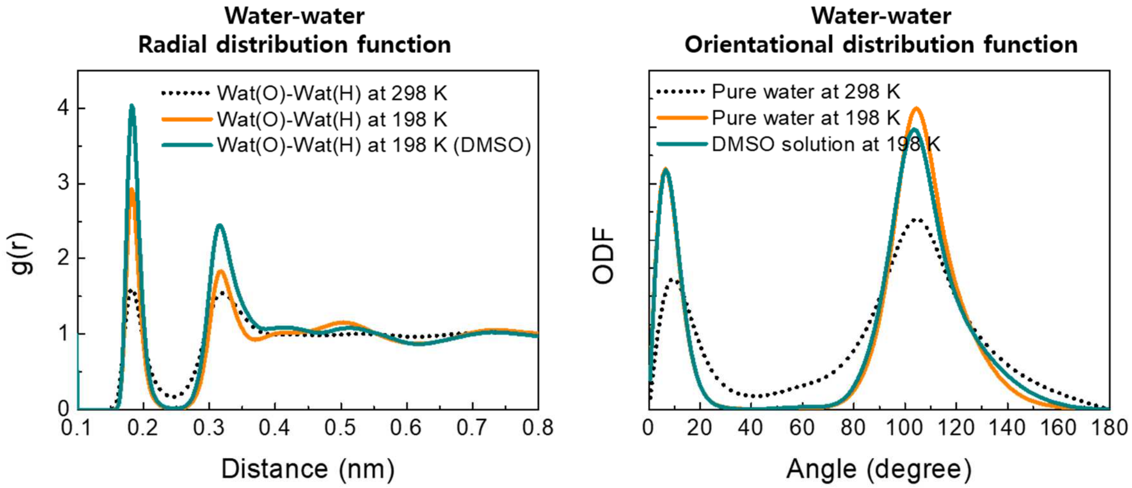

Figure S4. Computed instantaneous frequency distribution of the OD stretch mode of HDO in DMSO cosolvent system (top) and its 2D population with frequency and distance between DMSO sulfur atom and water deuterium atom. The color scale indicates the number of molecules within the distribution.

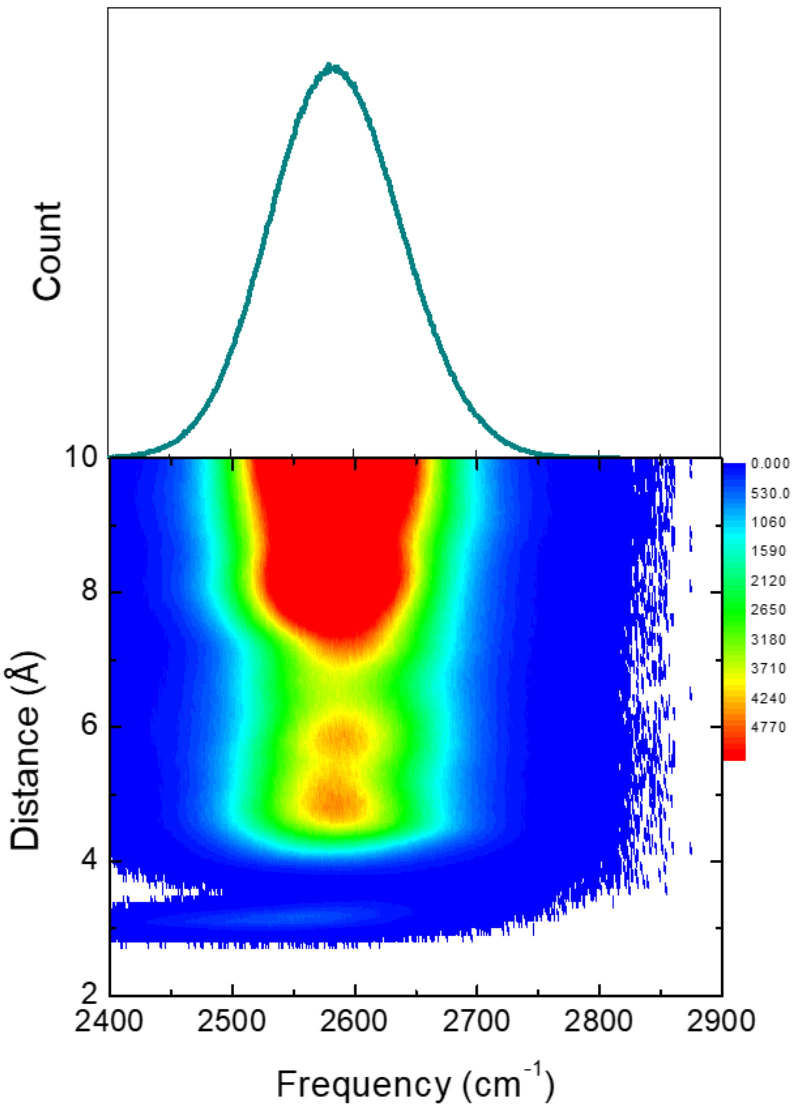

Figure S5. Computed instantaneous frequency distribution of the OD stretch mode of HDO in the first hydration shell of DMSO (top) and its "zoomed-in" effective 2D population as a function of frequency and distance between DMSO sulfur atom and water deuterium atom for waters within the first solvation shell of DMSO.

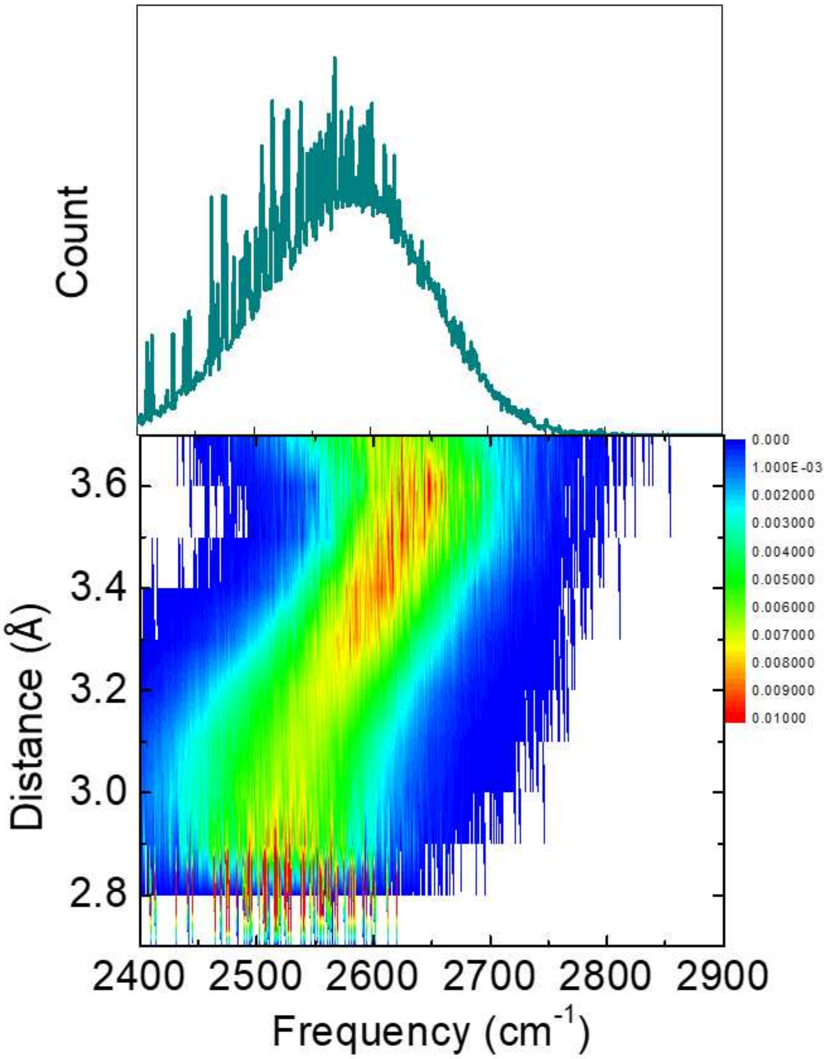

Figure S6. Histogram of the tetrahedral order parameter distribution of water molecules within the first solvation shell of DMSO (blue) as well as in the bulk (red) from the 33 wt% DMSO solution at 198 K and pure water 196K (yellow). A cutoff distance of 4.5 Å (S_{DMSO} – O_{Wat}) was used to assign waters to the first solvation shell of DMSO. In the 33 wt% DMSO simulations, 29% of water molecules were within first solvation shell of DMSO.

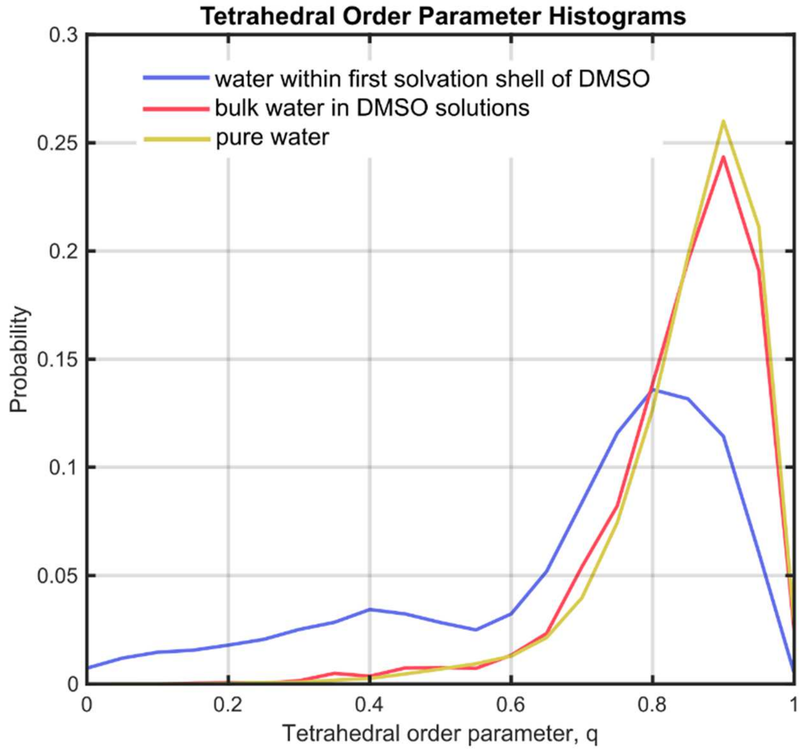

Table S1. Gaussian fit peak parameters of the OD stretch mode of HDO at different DMSO concentrations (Fig. 2). Red-colored parameters correspond to newly observed high frequency shoulder peak in DMSO solutions (shaded peak in Figure 2, main text).

DMSO concentration (wt%)		Parameters		
		Population (%)	FWHM (cm^{-1})	Center peak frequency (cm^{-1})
0	Peak1	20.08176	36.50514	2408.349
	Peak2	58.68859	21.79244	2430.306
	Peak3	21.22966	39.28465	2452.082
11	Peak1	13.36187	27.93606	2408.219
	Peak2	44.45823	21.60272	2428.392
	Peak3	14.17297	28.28665	2449.154
	Peak4	28.00693	120.0179	2455.056
22	Peak1	14.06079	33.42657	2410.334
	Peak2	36.09681	23.04348	2429.865
	Peak3	11.21833	26.24199	2452.497
	Peak4	38.62407	119.065	2457.479
33	Peak1	17.93015	42.28722	2407.259
	Peak2	24.77576	25.72168	2429.29
	Peak3	15.07715	35.20019	2452.136
	Peak4	42.21693	113.8221	2478.774

Table S2. Vibrational map formula and parameters to generate instantaneous frequency distributions (Figures 3 and S4) from MD snapshots (ref. 30 in main text). The parameters represent the center frequency (ω_0) and l_1- l_3 the electrostatic potentials at Sites 1-3 as indicated in the table respectively.

$$\Delta\omega(\phi) = \omega(\phi) - \omega_0 = \sum_{m=1}^{n} l_m \phi(\mathbf{R}_m)$$

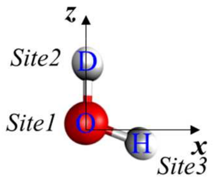

Parameters	
ω_0	2812.5 cm^{-1}
l_1	-0.022013 e
l_2	0.028628 e
l_3	-0.0066150 e
